# Effect of Dietary Inclusion of House Cricket (*Acheta domesticus*) Meal on Carcass Characteristics and Meat Quality in Rabbits

**DOI:** 10.3390/ani16162596

**Published:** 2026-08-19

**Authors:** Alda Quattrone, Naouel Feknous, Nour Elhouda Fehri, Gabriele Brecchia, Michela Contò, Majlind Sulçe, Giulio Curone, Gerald Muça, Daniele Vigo, Laura Menchetti, Olimpia Barbato, Egon Andoni, Valentina Serra, Sabrina Di Giovanni, Francesca Falcinelli, Grazia Pastorelli, Marta Castrica, Sebastiana Failla

**Affiliations:** 1 Department of Veterinary Medicine and Animal Sciences, University of Milan, Via dell’Università 6, 26900 Lodi, Italy; alda.quattrone@unimi.it (A.Q.); giulio.curone@unimi.it (G.C.); daniele.vigo@unimi.it (D.V.); valentina.serra@unimi.it (V.S.); grazia.pastorelli@unimi.it (G.P.); 2 Biotechnologies Related to Animal Reproduction, Institute of Veterinary Sciences, University Blida 1, B.P 270, Road of Soumaa, Blida 9000, Algeria; naouel_feknous@univ-blida.dz; 3 Department of Veterinary Medicine, University of Perugia, Via San Costanzo 4, 06126 Perugia, Italy; gabriele.brecchia@unipg.it (G.B.); olimpia.barbato@unipg.it (O.B.); francesca.falcinelli@collaboratori.unipg.it (F.F.); 4 Consiglio per la Ricerca in Agricoltura e l’Analisi Dell’Economia Agraria (CREA), Centro di Ricerca Zootecnia e Acquacoltura, Research Centre for Animal Production and Aquaculture, Via Salaria 31, 00015 Rome, Italy; michela.conto@crea.gov.it (M.C.); sabrina.digiovanni@libero.it (S.D.G.); sebastiana.failla@crea.gov.it (S.F.); 5 Faculty of Veterinary Medicine, Agricultural University of Tirana, Koder Kamez, 1029 Tirana, Albania; msulce@ubt.edu.al (M.S.); gmuca@ubt.edu.al (G.M.); eandoni@ubt.edu.al (E.A.); 6 School of Biosciences and Veterinary Medicine, University of Camerino, Via Circonvallazione 93/95, 62024 Matelica, Italy; laura.menchetti@unicam.it; 7 Department of Life Science, Health and Health Professions, Università degli Studi “Link Campus University”, Via del Casale di S. Pio V 44, 00165 Rome, Italy; m.castrica@unilink.it

**Keywords:** *Acheta domesticus*, insect meal, rabbit meat, carcass traits, fatty acid profile, sustainable animal nutrition

## Abstract

The search for sustainable feed ingredients has increased interest in insect-derived proteins for livestock production. This study evaluated the effects of replacing 3% soybean meal with house cricket (*Acheta domesticus*) meal on carcass traits, meat quality, proximate composition, and fatty acid profile of growing rabbits. Twenty-four weaned New Zealand White rabbits were assigned to either a control (CNT) or a cricket meal diet (ADM) from 35 to 85 days of age. Physical meat quality was evaluated in the *Longissimus thoracis et lumborum* (LTL), while proximate composition and fatty acid profile were determined in LTL and thigh muscles. Carcass performance was not affected by dietary treatment, whereas the cricket meal diet increased adiposity, resulting in greater scapular and perirenal fat deposition (*p* < 0.001) and a higher fat proportion in the hind leg (*p* = 0.011). Physical meat quality traits were unaffected, except for higher cooking loss in ADM-fed rabbits (*p* = 0.039). Cricket meal increased crude fat and modified the fatty acid composition by increasing n-6 PUFA (*p* = 0.013) and reducing n-3 PUFA (*p* < 0.001), resulting in a less favorable n-6/n-3 ratio. Overall, cricket meal modified lipid deposition and fatty acid composition without compromising carcass performance, although its greater lipid contribution should be considered when interpreting these effects.

## Simple Summary

The use of insect-derived ingredients in animal feeding has attracted increasing interest as a potential alternative to conventional protein sources. However, information on the effects of house cricket (*Acheta domesticus*) meal on rabbit meat quality is still limited. This study evaluated the effects of partially replacing soybean meal with house cricket meal on carcass characteristics, physical meat quality, proximate composition, and fatty acid profile in growing rabbits. The dietary inclusion of cricket meal did not affect carcass yield or the main physical characteristics of the meat, indicating that partial replacement of soybean meal did not adversely influence these traits. However, rabbits fed the cricket-based diet showed greater carcass adiposity and higher intramuscular fat content. The fatty acid composition of the meat was also modified, with increased proportions of n-6 polyunsaturated fatty acids and reduced proportions of n-3 polyunsaturated fatty acids compared with rabbits fed the control diet. These findings indicate that house cricket meal can be included as a partial substitute for soybean meal in rabbit diets without negatively affecting carcass characteristics or the main physical properties of the meat. Nevertheless, the associated changes in meat fatty acid composition highlight the importance of considering the lipid composition of insect-based ingredients when formulating rabbit diets.

## 1. Introduction

The increasing global demand for animal-derived foods, together with concerns regarding environmental sustainability and the dependence of livestock production on conventional protein sources such as soybean meal and fishmeal, has intensified the search for alternative feed ingredients capable of supporting efficient and sustainable animal production [1,2,3]. Among the proposed alternatives, insects have attracted considerable interest because of their high nutritional value, efficient feed conversion, and relatively low environmental footprint compared with conventional protein sources [4,5,6]. In addition to providing high-quality proteins and lipids, insect-derived products are also rich in vitamins, minerals, and other bioactive compounds that support their use in animal feeding [4,7,8]. Although their nutritional composition varies among species and according to rearing conditions, several edible insects have emerged as promising protein sources for livestock [9,10]. Among these, the house cricket (*Acheta domesticus*) features a high crude protein content and a favorable amino acid profile, making it a promising ingredient for monogastric diets [11,12,13].

The growing interest in insects as feed ingredients has been accompanied by progressive regulatory developments within the European Union. Processed animal proteins (PAPs) derived from several insect species, including *Acheta domesticus*, are currently authorized for use in aquaculture, poultry, and pig feeds under specific legislative provisions [14,15,16,17]. In contrast, under the European feed ban framework established by Regulation (EC) No 999/2001 [18], the use of insect PAPs in rabbit diets remains prohibited. As a result, research on insect-derived ingredients in rabbits remains scarce and is currently confined to experimental studies.

Rabbits represent an efficient livestock species owing to their high reproductive performance, rapid growth, and favorable feed conversion efficiency [19]. Rabbit meat is also appreciated for its high protein content, low fat concentration, and favorable lipid profile [20]. Nevertheless, the increasing cost of conventional feed ingredients continues to challenge the sustainability of rabbit production systems, stimulating interest in alternative protein sources [21]. Although insects have been extensively investigated in aquaculture, poultry, and pig nutrition, studies evaluating their use in rabbits remain relatively limited [22,23,24,25,26,27,28,29,30] and have primarily focused on black soldier fly (*Hermetia illucens*), yellow mealworm (*Tenebrio molitor*), and silkworm (*Bombyx mori*) products [30,31,32,33,34]. Overall, moderate dietary inclusion levels have generally been well tolerated without detrimental effects on productive performance, although variable responses have been reported for carcass characteristics and meat quality. Because insects are also a substantial source of lipids, their inclusion can significantly influence the fatty acid composition and nutritional quality of rabbit meat, which is highly responsive to dietary lipid sources [35].

To date, only one study has evaluated the use of *Acheta domesticus* meal in rabbit diets. Volek et al. [32] demonstrated that cricket meal can successfully replace soybean meal without detrimental effects on growth performance, nutrient digestibility, or nitrogen balance. However, information regarding the effects of this protein source on carcass characteristics and meat quality have not yet been investigated. Given the growing demand for sustainable protein alternatives and the lack of comprehensive data for this species, further investigation is essential. To address this knowledge gap, the present study extends previous research by evaluating the impact of partially replacing dietary soybean meal with *Acheta domesticus* meal on the carcass traits, physical meat quality, proximate composition, and detailed fatty acid profile of growing rabbits, with particular attention to the different contribution of carbohydrates from the plant-based ingredient and lipids from the insect-derived ingredient to dietary energy supply, and to their potential influence on the nutritional quality of meat.

## 2. Materials and Methods

### 2.1. Animals and Diets

The experimental trial was conducted at the Faculty of Veterinary Medicine of the Agricultural University of Tirana. The experimental protocol was approved by the Ministry of Agriculture and Rural Development, National Authority of Veterinary and Plant Protection of Albania (Protocol No. 824/2021). All experimental procedures were performed in accordance with national and international guidelines for the care and use of animals in research. Particular attention was paid to minimizing animal suffering and reducing the number of animals used while ensuring the reliability of the experimental results. Animal health status and welfare conditions were monitored daily throughout the experimental period by the attending veterinarian.

A total of 24 weaned New Zealand White rabbits of mixed sex (18 males and 6 females, equally distributed between the two dietary treatments, with 9 males and 3 females per treatment) were enrolled in the study at 35 ± 2 days of age. Rabbits were individually housed in conventional wire cages until they were slaughtered at 85 ± 2 days of age. Environmental conditions were maintained at a temperature ranging from 18 to 21 °C and a relative humidity of approximately 60%, under a controlled photoperiod of 16 h light and 8 h dark.

At weaning, rabbits were randomly assigned to one of two dietary treatments: (i) a control group (CNT; *n* = 12), fed a standard commercial diet in which soybean meal served as the main protein source, and (ii) a cricket meal group (ADM; *n* = 12), fed a diet containing adult house cricket (*Acheta domesticus*) meal, the ADM diet incorporated the cricket meal at a 3% inclusion rate. The cricket meal was supplied from a commercial producer (Nutrinsect S.r.l., Montecassiano, MC, Italy). Both diets were formulated to be isoenergetic and isonitrogenous and to meet the nutritional requirements of growing rabbits according to current feeding recommendations [35]. However, the diets were not strictly isolipidic. The crude fat content of the protein sources was 2.17 g/100 g for soybean meal and 18.54 g/100 g for *Acheta domesticus* meal (Table S1), contributing to a higher analyzed ether extract (EE) content in the ADM diet than in the CNT diet (2.64 vs. 2.08 g/100 g, respectively; Table 1).

The ingredient composition of the experimental diets is presented in Table 2, whereas their analyzed chemical composition and fatty acid profile are reported in Table 2. Detailed information on the chemical composition, amino acid profile, and fatty acid composition of soybean meal and *Acheta domesticus* meal is provided in Supplementary Table S1.

Throughout the experimental period, rabbits received restricted daily feed allowances, which were progressively increased according to age from 90 to 140 g/day. Fresh drinking water was available ad libitum throughout the study.

### 2.2. Pellet Analysis

Representative samples of each pelleted diet were collected at the farm, vacuum-packed, transported to the laboratory, and finely ground to obtain a homogeneous material for chemical analyses.

Dry matter (DM), ash, crude protein (CP), ether extract (EE), and crude fiber (CF) were determined according to AOAC International procedures (AOAC 934.01, 942.05, 984.13, 920.39, and 978.10, respectively). Crude protein was calculated as nitrogen (N) × 6.25 using the Kjeldahl method. Digestible Energy (DE) was estimated according to De Blas and Mateos [35], using the equation:

DE kcal/kg = 1801 + (7.10 × CP) + (12.01 × EE) + (5.59 × Nitrogen-free extract).

Fiber fractions, including neutral detergent fiber (NDF), acid detergent fiber (ADF), and acid detergent lignin (ADL), were determined according to Van Soest et al. [36]. Chitin content was determined using a sequential acid–alkali extraction procedure involving demineralization and deproteinization steps, as previously described by Psarianos et al. [37]. All analytical results were expressed as g/100 g of pellet.

The fatty acid composition of the experimental diets was determined following lipid extraction and transesterification of fatty acids to their corresponding methyl esters (FAME), which were subsequently quantified by gas chromatography with flame ionization detection (GC-FID), following the same analytical procedure described for meat samples (Section 2.5.2).

### 2.3. Carcass Traits and Performance

At 85 days of age, all rabbits were slaughtered in an authorized slaughterhouse in accordance with Council Regulation (EC) No. 1099/2009. After slaughtering, carcasses were manually skinned and eviscerated, and the distal portion of the hind limbs was removed during dressing. The dressed carcasses were then stored under refrigerated conditions at 4 °C for 24 h. After chilling, the head and non-edible organs, including the lungs, thymus, and esophagus, were removed to determine the chilled carcass weight (CCW). Following this, the weights of the liver (LvW), scapular fat (SFaW), and perirenal-pelvic fat (PFaW) were individually recorded.

Carcasses were subsequently divided into three anatomical regions according to the guidelines of the World Rabbit Science Association [38]: the fore part (FLP), corresponding to the anterior portion of the carcass; the thoracic cage part (TCP), including the thoracic, lumbar, and abdominal regions; and the hind part (HPP), corresponding to the posterior portion. The weight of each portion was recorded and expressed as a percentage of chilled carcass weight. The *Longissimus thoracis et lumborum* (LTL) muscle was excised from each carcass and used for the evaluation of physical and chemical meat quality traits, including pH, color coordinates, drip loss, cooking loss, proximate composition, and fatty acid profile. In addition, the right hind leg was dissected into its main tissue components to determine the relative proportions of muscle, bone, and the combined fraction of intermuscular fat and connective tissue, thereby providing the assessment of carcass tissue distribution.

For chemical analyses, thigh meat (THM) samples were obtained by pooling the main hind limb muscles (*Biceps femoris*, *Semimembranosus*, and *Semitendinosus)*. Muscle samples were minced under refrigerated conditions (4 °C) to minimize temperature increase and limit oxidative alterations prior to subsequent analytical determinations.

### 2.4. Physical Analysis

#### 2.4.1. pH

Immediately after dissection, 1 g sample of LTL muscle was homogenized in a NaCl solution (0.1 mol/L, pH 7). Homogenization was performed using an Ultra-Turrax homogenizer (T25 digital ULTRA-TURRAX^®^, IKA, Staufen, Germany) for 30 s at 6000 rpm. The pH was measured using a digital pH meter equipped with automatic temperature compensation (XS Instrument Serie 80 PC80, Giorgio Bormac S.r.l., Carpi, MO, Italy). The final pH value was calculated as the average of two consecutive measurements obtained from two aliquots of the same sample.

#### 2.4.2. Water-Holding Capacity and Cooking Loss

Water-Holding Capacity (WHC) of the LTL muscle was determined according to the method described by Kauffman et al. [39], with minor modifications. Briefly, 1 g of finely chopped meat was placed between two filter paper discs and subjected to a 1 kg load for 5 min. After compression, the sample was reweighed, and the weight difference was expressed as percentage of water loss. All determinations were performed in duplicate.

Cooking loss was evaluated following the procedure described by Honikel [40]. Two weighed portions of the LTL muscle (excised from the right and left sides at the same anatomical location) were vacuum sealed in plastic bags under light vacuum conditions and cooked in a water bath at 75 °C for 20 min. After cooling in an ice bath, the samples were reweighed, and cooking loss was calculated as the average value of the two portions and expressed as a percentage of the initial sample weight.

#### 2.4.3. Color

Color measurements were performed on the LTL muscle using the CIELAB color system (International Commission on Illumination CIE) to determine lightness (L*), redness (a*), and yellowness (b*) [41]. The LTL muscle was first sectioned longitudinally and exposed to ambient air for 30 min to allow blooming prior to analysis. Measurements were obtained under D65 illuminant conditions (6504 K daylight) using a Konica Minolta CM-3600d spectrophotometer (Konica Minolta Sensing Inc., Osaka, Japan) operating with d/8◦ geometry. Color values were recorded as the mean of four distinct measurements per sample.

### 2.5. Chemical Analysis

#### 2.5.1. Proximate Composition

The proximate composition of LTL and THM samples was determined according to AOAC procedures [42] for dry matter (DM; method 950.46), crude fat (method 954.02), ash (method 942.05), and crude protein (method 990.03). Immediately after dissection, 8 g of sample was used for DM determination by oven drying at 102 °C for 24 h. Ash content was subsequently determined on the same samples by incineration at 540 °C for 8 h. Crude fat was analyzed using the Soxhlet extraction method with diethyl ether (Tecator Soxtec System HT 1043 extraction unit, Gemini, Apeldoorn, Sweden). Crude protein content (N × 6.25) was measured using the Kjeldahl method with a Tecator Digestion System and a Kjeltec Auto 1030 Analyzer (Tecator, Apeldoorn, Sweden).

#### 2.5.2. Fatty Acid Profile

Total lipids were extracted from 10 g of LTL muscle and minced THM using a chloroform:methanol mixture (2:1, *v*/*v*). The extracted fat was diluted in hexane and methylated following the IUPAC procedure [43] using a 2 M methanolic KOH solution to obtain fatty acid methyl esters (FAMEs). For fatty acids quantification, 1 μL of FAME sample was injected into a gas chromatograph (GC 6890N, Agilent Technologies Inc., Santa Clara, CA, USA) equipped with a flame ionization detector (FID) and a CP-Sil 88 fused-silica capillary column (100 m × 0.25 mm internal diameter, 0.20 μm film thickness; Agilent Technologies, Santa Clara, CA, USA). The specific gas chromatographic conditions were those described in detail by Failla et al. [44]. Nonadecanoic acid (19:0) was added as an internal standard to the samples prior to fatty acid extraction. FAMEs were identified by comparing their peak retention times with those of the Supelco 37 Component FAME Mix and the docosapentaenoic acid standard (Sigma-Aldrich Merck, Darmstadt, Germany). Fatty acid concentrations were quantified and expressed as mg/100 g of meat. Different classes of fatty acids, including saturated fatty acids (SFA), monounsaturated fatty acids (MUFA), total polyunsaturated fatty acids (PUFA), n-6 PUFA, n-3 PUFA, and the n-6/n-3 ratio, were calculated and expressed both as percentage of total FAMEs and as mg/100 g of meat using nonadecanoic acid as an internal standard. Furthermore, the atherogenic index (AI), thrombogenic index (TI) [45], and the PUFA to SFA ratio (P/S) [46] were calculated.

### 2.6. Statistical Analysis

All statistical analyses were performed using the statistical software package SAS v. 9.4 (SAS Institute Inc., Cary, NC, USA). Data were initially checked for normal distribution and homogeneity of variances prior to analysis.

Carcass traits and physical quality parameters of LTL were analyzed using a one-way analysis of variance (ANOVA).

For chemical composition and fatty acid profile, a linear mixed model including animal as a random effect was initially evaluated to account for the repeated measurements obtained from the two muscles of the same rabbit. Model selection was based on the estimated animal variance, the Intraclass Correlation Coefficient (ICC), and the Akaike Information Criterion (AIC). Since the estimated animal variance was generally negligible and the mixed model did not provide a meaningful improvement in model fit, the more parsimonious two-way ANOVA was retained for the final analyses.

As the experimental population included both males and females, a preliminary exploratory analysis was performed to assess the potential effect of sex on the investigated traits (Supplementary Tables S2–S4). No significant sex-related differences were detected. Given the numerical imbalance between males and females, sex and its interactions with the other fixed factors were therefore excluded from the final models to avoid unnecessary model complexity. This choice was also supported by previous findings in New Zealand White rabbits showing no appreciable sex-related differences at comparable ages as reported by Eniwaiye et al. [47]. Accordingly, animals were treated as a mixed-sex population in the subsequent analyses.

Chemical analyses of meat, including proximate composition and fatty acid profile, were therefore analyzed using a two-way ANOVA including the interaction between dietary treatment and muscle type, according to the following model:

Y*_ijk_* = µ + D_i_ + M_j_ + (D × M)_ij_+ e_ijk_ (1)

where Y*_ijk_* is the observed variable, µ is the overall mean, D*_i_* is the fixed effect of diet (_i_ = CNT or ADM, corresponding to the control diet and diet containing cricket meal), M*_j_* is the fixed effect of muscle type (_j_ = LTL or THM), (D × M)*_ij_* is the interaction between diet and muscle, and e_ijk_ is the residual error.

When significant effects were detected, means were separated using Tukey’s multiple comparison test. Statistical significance was declared at *p* < 0.05.

Finally, carcass performance data (CCW, PFaW, SFaW), physical traits (cooking loses, L*, a*, b*), and chemical variables on LTL (protein and fat%, 16:0, 18:0, 17:0, 16:1, 18:1cis9, 18:2 n-6, 18:3 n-3, 20:4 n-6, 20:5 n-3, 22:5 n-3, 22:6 n-3) were subjected to an exploratory multivariate analysis by Principal Component Analysis (PCA). Prior to PCA, the twenty variables were log-transformed to minimize potential scale-dominance and carry-over effects associated with variables characterized by different numerical ranges and magnitudes. Since CIELAB a* values were negative, their absolute values were used exclusively for the logarithmic transformation to allow their inclusion in the multivariate analysis. The transformed dataset was subsequently centered and standardized before multivariate analysis. Principal components were used to explore sample distribution patterns and variable contributions associated with dietary treatment. The complete PCA loadings for all variables are provided in Supplementary Table S5 to facilitate interpretation of their contribution to the principal components.

## 3. Results

The preliminary analysis did not detect significant sex-related differences for any of the productive, carcass, meat quality, proximate composition, or fatty acid profile traits considered (Supplementary Tables S2–S4). Therefore, the subsequent results are presented for the mixed-sex population.

### 3.1. In Vivo and Slaughter Performance

Results for live weight, carcass traits, and tissue composition are presented in Table 3. Rabbits assigned to the two dietary treatments had comparable body weights at weaning (774 g on average), confirming the initial homogeneity of the experimental groups. Dietary inclusion of *Acheta domesticus* meal did not significantly affect slaughter weight (*p* > 0.05), chilled carcass weight (CCW), or liver weight (LvW; *p* = 0.158). CCW was numerically higher in rabbits fed the ADM diet than in the control group, with an average increase of approximately 74 g; however, this difference did not reach statistical significance (*p* > 0.079).

In contrast, fat deposition was markedly influenced by dietary treatment, as rabbits receiving the ADM diet showed significantly greater scapular fat weight (SFaW) and perirenal–pelvic fat weight (PFaW) than those in the CNT group (*p* < 0.001 for both traits). These differences should be interpreted in the context of the dietary formulation, as the ADM and CNT diets were not strictly isolipidic and differed in both the amount and composition of dietary lipids. The relative proportions of the main carcass cuts were not influenced by the diet, as no significant differences were observed for foreleg part (FLP), thoracic cage part (TCP), or hind part proportion (HPP) (*p* > 0.05). However, carcass tissue composition within the hind leg was significantly modified by the house cricket supplementation. Rabbits fed the ADM diet exhibited a lower proportion of hind leg muscle (HLMeat) (*p* = 0.047) and a higher proportion of hind leg fat and associated tissues (HLFat) (*p* = 0.011) compared with the CNT group, whereas the proportion of hind leg bone (HLBone) remained unaffected (*p* = 0.705).

### 3.2. Physicochemical Characteristics of Rabbit Meat

The physical characteristics of the *Longissimus thoracis et lumborum* (LTL) muscle are reported in Table 4. The ADM diet did not affect the ultimate pH of the LTL muscle (*p* = 0.152). Likewise, the water-holding capacity (WHC) remained unchanged (*p* = 0.980).

A significant effect was observed for cooking loss, which was higher in rabbits fed ADM than in the CNT group (28.65 vs. 26.62%; *p* = 0.039).

Meat color was not affected by the dietary treatment, as no significant differences were observed for lightness (L*), redness (a*), or yellowness (b*) (*p* > 0.05). The negative a* values observed in both groups are consistent with the low myoglobin concentration typically reported in rabbit meat, further confirming the absence of dietary effects on muscle pigmentation.

**Figure 1:**
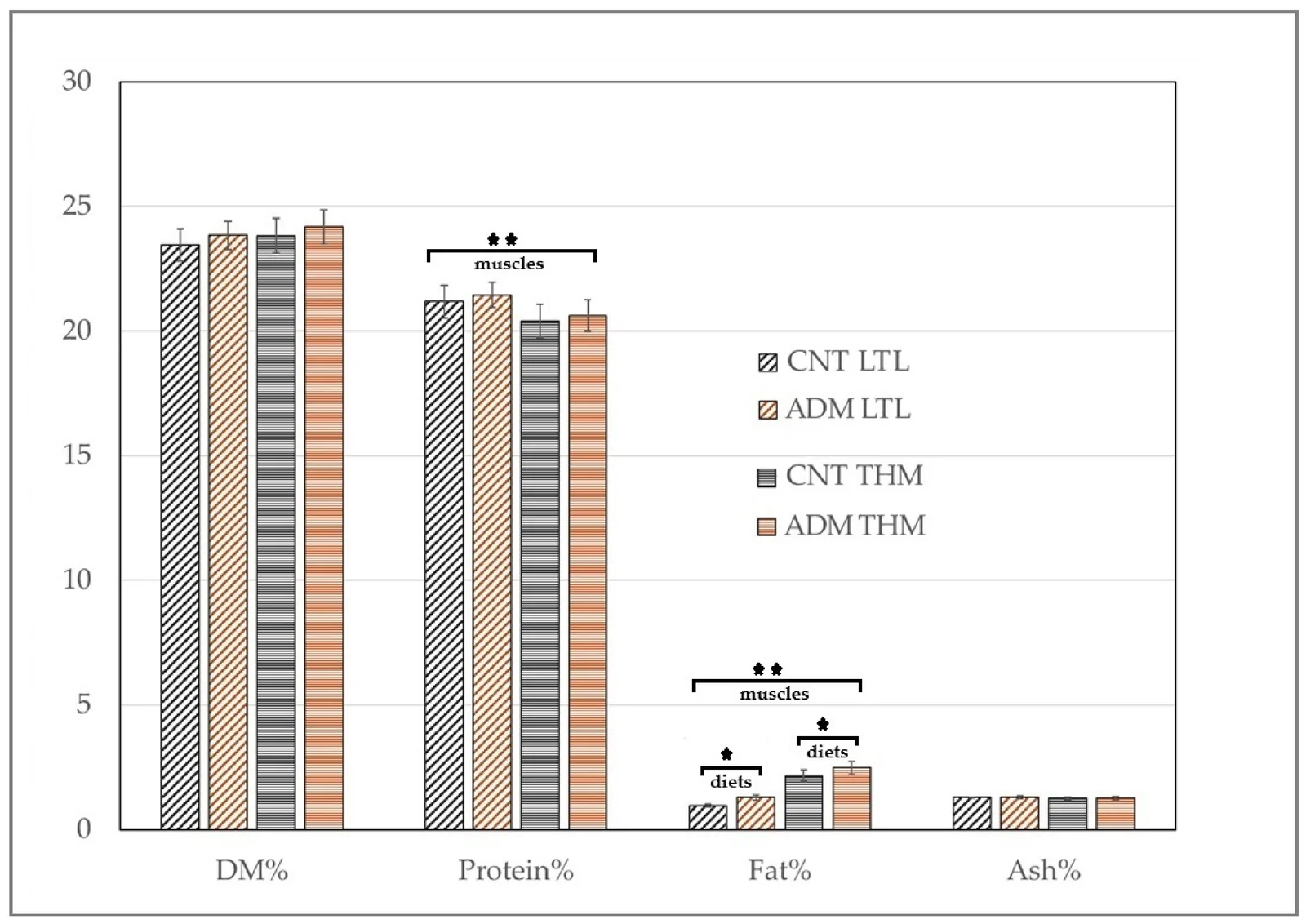
Proximate composition of the *Longissimus thoracis et lumborum* (LTL) and thigh muscles (THM) of rabbit fed the two experimental diets. CNT: control diet; ADM: diet containing *Acheta domestics* meal; DM: dry matter. Significant differences between diets and between muscles are reported as * *p* < 0.05; ** *p* < 0.01. The absence of symbols indicates no significant differences (*p* > 0.05).

The proximate composition of the LTL and THM muscles is shown in Figure 1. Dry matter and ash were not affected by either diet and muscles. In contrast, protein content was significantly influenced by muscle type (*p* < 0.01), with higher values observed in the LTL muscle than in the THM muscle, regardless of dietary treatment. Fat content was affected by diet (*p* < 0.05) and muscle type (*p* < 0.01); rabbits fed the ADM diet exhibited higher fat percentage than those receiving the CNT diet. Moreover, fat content was substantially higher in THM (2.17 and 2.39% in CNT and ADM, respectively, for the two groups) than in LTL (0.95 and 1.07%, respectively).

### 3.3. Fatty Acid Profile of Rabbit Meat

The fatty acid profile of rabbit meat was significantly influenced by the dietary treatment (Table 5). Saturated fatty acids (SFA) and *cis*-monounsaturated fatty acids (MUFAc) were not significantly affected by dietary treatment (*p* > 0.05). Among the individual saturated fatty acids, palmitic acid (16:0) was the predominant SFA, accounting for approximately 27–28% of total FAME, followed by stearic acid (18:0), which ranged between 7.7 and 8.8% (Table S4). Rabbits fed the ADM diet exhibited lower proportions of odd- and branched-chain fatty acids (OBCFA; *p* < 0.001) and higher proportions of *trans*-monounsaturated fatty acids (MUFAt; *p* < 0.001) than those fed the CNT diet. These differences reflected changes in several individual fatty acids, as detailed in Table S4.

Dietary treatment also affected the polyunsaturated fatty acid fraction (PUFA). A significant increase in n-6 PUFA was observed in ADM-fed rabbits (*p* = 0.013), whereas n-3 PUFA decreased (*p* < 0.001), resulting in a marked increase in the n-6/n-3 ratio (*p* < 0.001). As detailed in Table S1, the increase in the n-6 fraction was mainly attributable to the higher proportion of linoleic acid (LA; 18:2 n-6), which increased from 17.92 to 20.72% in the LTL muscle and from 20.49 to 22.53% in the THM; no difference was reported for arachidonic acid. Conversely, the reduction in the n-3 fraction was primarily associated with lower concentrations of α-linolenic acid (ALA;18:3 n-3), docosapentaenoic acid (DPA; 22:5 n-3), and docosahexaenoic acid (DHA; 22:6 n-3).

Muscle type significantly affected all major fatty acid classes except MUFAc (*p* = 0.223). In general, THM exhibited higher proportions of SFA, MUFAt, and n-6 PUFA, whereas LTL showed greater levels of n-3 PUFA and consequently a lower n-6/n-3 ratio.

The dietary treatment had limited effects on the nutritional lipid quality indices. The atherogenicity (AI) and thrombogenicity (TI) indices remained substantially unchanged between the experimental groups. Conversely, the polyunsaturated-to-saturated fatty acid (P/S) ratio was significantly higher in rabbits fed the ADM diet. This effect was mainly attributable to the greater proportion of linoleic acid (18:2 n-6) observed in this group.

The concentrations of the major fatty acid classes expressed as mg/100 g of meat are reported in Table 6. Dietary treatment significantly affected most unsaturated fatty acid classes. Rabbits fed the ADM diet showed higher concentrations of MUFAc (*p* = 0.011), MUFAt (*p* = 0.012), n-6 PUFA (*p* = 0.009), and total PUFA (*p* = 0.028) than those fed the CNT diet. In contrast, n-3 PUFA concentrations were lower in the ADM group (*p* = 0.006).

Although the relative proportion of SFA was not significantly affected by dietary treatment, SFA concentrations expressed on a meat basis were numerically higher in ADM-fed rabbits, although the difference did not reach statistical significance (*p* = 0.055). Conversely, OBCFA were unaffected when expressed as mg/100 g of meat (*p* = 0.820), despite the lower relative proportions observed in ADM rabbits. The discrepancy between relative and absolute values is consistent with the higher lipid content recorded in ADM-fed rabbits. Likewise, the higher concentrations of most fatty acid classes observed in THM compared with LTL paralleled the greater fat content of this muscle.

Muscle type significantly influenced all fatty acid classes (*p* < 0.001). Compared to the LTL muscle, the THM contained greater amounts of SFA, MUFA, and PUFA. In contrast, the dietary effects were consistent across both muscles, with no evidence of different responses between the anatomical sites.

The significant increase in n-6 PUFA and the concurrent reduction in n-3 PUFA observed on a percentage basis (Table 5) were also confirmed when fatty acid classes were expressed as mg/100 g of meat. In particular, ADM-fed rabbits showed higher concentrations of n-6 PUFA and lower concentrations of n-3 PUFA in both muscles, resulting in a greater absolute content of total PUFA but a less favorable balance between the two polyunsaturated fatty acid families.

### 3.4. Results of Principal Component Analysis

**Figure 2:**
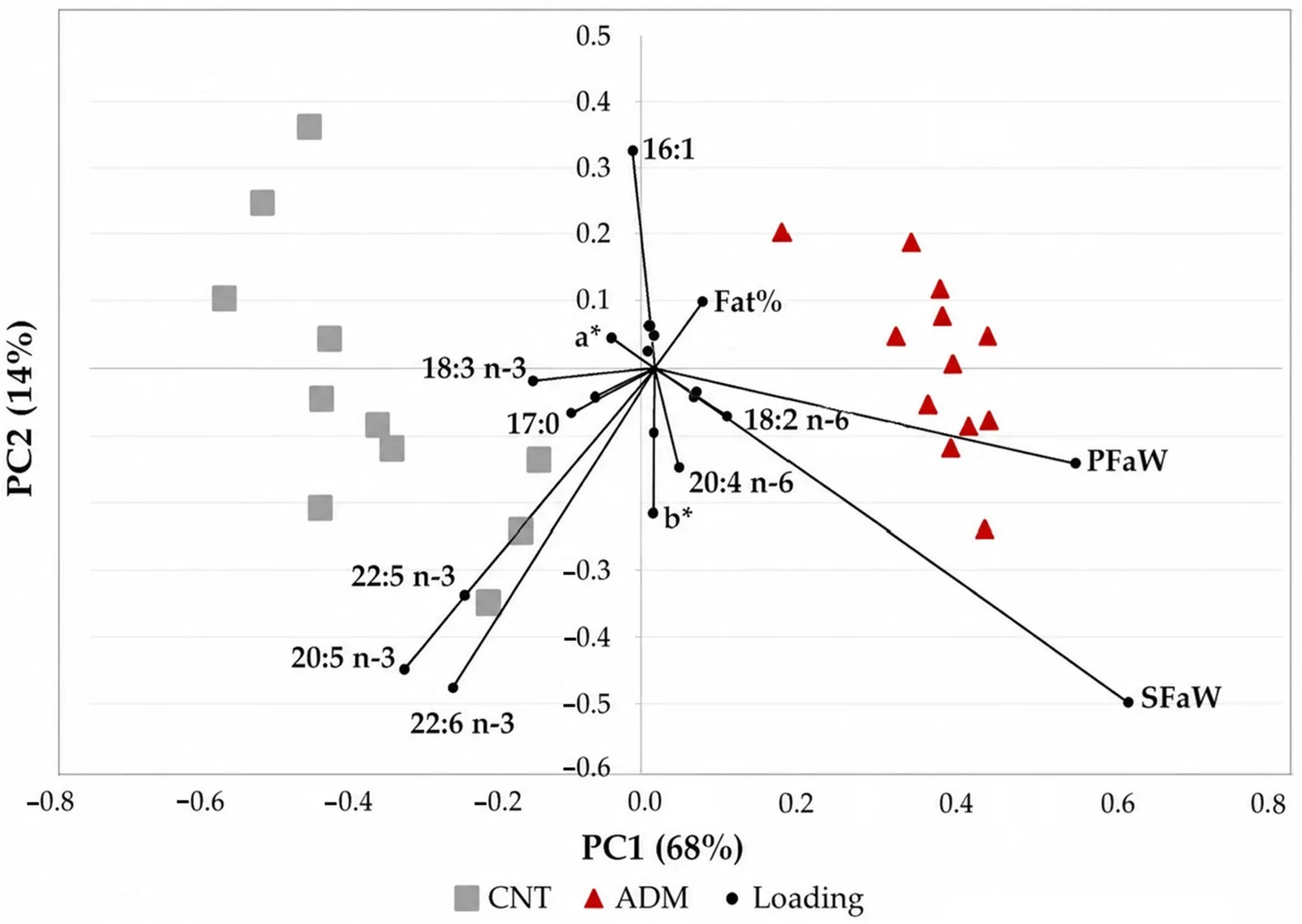
Principal Component Analysis (PCA) score and loading plots based on carcass traits, physical parameters, and some fatty acids of *Longissimus thoracis et lumborum* muscle (LTL) of rabbits fed the control diet (CNT) and the ADM diet containing *Acheta domesticus* meal. The PCA was performed using log-transformed and standardized variables to reduce scale-related effects among parameters. PC1 and PC2 explained 68% and 14% of the total variance, respectively. Variables with loadings close to zero and therefore not labelled in the loading plot were CCW, LTL crude protein, cooking loss, L, 16:0, 18:0, and 18:1 cis-9.

To minimize the effect of variable magnitude and improve the comparability of parameters expressed on different scales, carcass traits, physical parameters, and fatty acid were log-transformed and standardized before PCA (Figure 2). The first two principal components explained 82% of the total variance. The score plot revealed a clear separation between rabbits fed the CNT and ADM diets.

According to the loading plot this separation was primarily associated with variables related to fat deposition and fatty acid composition. The complete loading values for all variables included in the PCA are reported in Supplementary Table S5. Specifically, ADM samples were strongly associated with greater fat depots, higher intramuscular fat content, and n-6 fatty acids. Conversely, CNT samples were characterized by higher relative proportions of 17:0 and principal n-3 fatty acids. Physical meat quality traits showed lower contributions to group discrimination.

## 4. Discussion

### 4.1. In Vivo and Slaughter Performance

The partial replacement of soybean meal with *Acheta domesticus* meal had limited effects on overall carcass performance, consistent with previous studies showing that insect-derived ingredients can replace conventional protein sources without adversely affecting carcass yield or the proportions of commercial cuts [30,47]. In the present study, although rabbits fed the ADM diet showed numerically higher chilled carcass weight, although the difference was not statistically significant, indicating that the dietary treatment did not substantially modify overall carcass development.

The main dietary effect was of cricket meal inclusion was the greater adipose tissue deposition, particularly in the scapular and pelvic regions, together with a higher fat proportion and a lower meat proportion in the hind leg. These findings only partially agree with those of Volek et al. [32], who reported improved growth performance following *Acheta domesticus* supplementation during the post-weaning period but did not evaluate carcass composition. Such discrepancies are likely related to the physiological stage of the animals. During the early growth phase, nutrients are preferentially directed towards protein accretion, whereas, as rabbits approach slaughter age, the efficiency of lean tissue deposition progressively declines, and adipose tissue becomes a more important determinant of body composition [48]. The greater adiposity observed in ADM rabbits may also be explained by the distinct nutritional profile of the cricket meal. Although the experimental diets were formulated to be isoenergetic and isonitrogenous, *Acheta domesticus* meal differs from soybean meal in both lipid content and fatty acid composition. In particular, *Acheta domesticus* provides a larger lipid fraction in addition to highly digestible protein [49]. Consequently, the relative contribution of macronutrients to energy supply differed between treatments, with a greater proportion of dietary energy being derived from lipids rather than carbohydrates in ADM rabbits. Since dietary fat can be incorporated into body lipid reserves more readily than carbohydrates, which require de novo lipogenesis before contributing to fat storage [50,51], differences in the form of dietary energy may influence body composition even when total energy intake remains comparable. Collectively, these findings suggest that cricket meal did not enhance carcass growth per se but rather promoted a modest increase in adiposity, redirecting nutrient utilization towards fat deposition without substantially altering carcass yield or anatomical distribution.

The absence of significant sex-related effects should be interpreted cautiously, given the unbalanced sex distribution and the limited number of females, and should not be taken as evidence that biologically relevant sex differences are absent. Nevertheless, the relatively young slaughter age may have limited the expression of sex-related differences, as the rabbits were slaughtered before reaching full sexual maturity. This interpretation is consistent with previous observations in New Zealand White rabbits showing broadly similar growth patterns between males and females up to approximately 84 days of age, with sex-related differences becoming more evident at later stages of growth [46].

### 4.2. Effect of the Experimental Diets on the Physical Characteristics of Rabbit Meat

The inclusion of *Acheta domesticus* meal did not significantly affect the ultimate pH, WHC or color parameters of the LTL muscle, indicating that the partial replacement of soybean meal with cricket meal did not compromise the main physical quality traits of rabbit meat. This finding is particularly relevant because meat color is a key determinant of consumer acceptance and is closely associated with post-mortem muscle metabolism, pH, and WHC [19]. Our results agree with those of Dalle Zotte et al. [52], who reported that insect-derived feed ingredients generally have limited effects on the physical quality of meat in poultry, pigs, and rabbits. Broader evidence from poultry research further supports these observations. In a recent review, Malematja et al. [53] reported that, although insect meals may induce small changes in meat redness and yellowness, these generally remain within the range of consumer acceptability and have little impact on other physical quality traits. Moreover, Mustafa et al. [54] found that dietary inclusion of *Acheta domesticus* meal affected meat quality in broiler chickens only at inclusion levels exceeding 12%, whereas lower inclusion rates had negligible effects on color and WHC. The available evidence therefore suggests that moderate levels of cricket meal inclusion are unlikely to substantially affect the physical quality of meat, although species-specific responses cannot be entirely excluded.

The only physical parameter affected by dietary treatment was cooking loss, which was slightly higher in rabbits fed the ADM diet. However, this finding should be interpreted cautiously, since the absence of concomitant changes in pH and drip loss suggests that the overall water-holding capacity of raw meat was largely preserved. Therefore, the increase in cooking loss is unlikely to reflect a generalized impairment of muscle protein functionality. Instead, it may partly reflect the slightly higher intramuscular fat content observed in ADM-fed rabbits, although the difference in fat content between groups was relatively small. Indeed, heat treatment simultaneously promotes water exudation and the release of melted lipids, which possess a relatively low melting point and can be lost alongside meat juices during cooking [54].

### 4.3. Proximate Composition and Fatty Acid Profile of Different Muscles

The higher crude fat percentage observed in the meat from ADM rabbits was consistent with the greater carcass adiposity described previously and appears to be a consequence of the different metabolic fate of nutrients rather than differences in total energy intake. The absence of substantial changes in muscle protein content further suggested that cricket meal did not impair protein accretion, and that the observed differences in meat composition were largely a consequence of the greater adiposity.

Muscle type exerted a greater influence than diet on proximate composition, with the THM muscle exhibiting a higher lipid content than the LTL. This difference is consistent with the predominantly oxidative metabolism of the THM, which is typically associated with greater intramuscular fat deposition than the predominantly glycolytic LTL [44,55]. Similar responses have been reported in rabbits fed diets containing other insect-derived ingredients, including *Hermetia illucens*, *Tenebrio molitor*, and *Bombyx mori*, where dietary inclusion was associated with increased or only marginally affected intramuscular fat deposition, whereas moisture, protein, and ash contents remained largely unchanged [33,56,57].

Dietary treatment modified the overall fatty acid profile of rabbit meat; however, the response differed among fatty acid classes. In particular, changes in the saturated fatty acid (SFA) fraction were limited. Despite the ADM diet containing slightly higher proportions of palmitic acid (16:0) and stearic acid (18:0), their concentrations in rabbit meat did not differ significantly from those of the control group. This finding is consistent with the metabolic origin of these fatty acids, particularly 16:0, which represents the main end-product of de novo lipogenesis and is therefore strongly regulated by endogenous metabolism rather than being exclusively determined by dietary intake. Similarly, 18:0 is not only supplied by the diet but also derives from elongation and subsequent metabolic transformations occurring within tissues [58,59]. Consequently, moderate differences in dietary SFA supply are unlikely to substantially affect their deposition in rabbit muscle. Importantly, the deposition of dietary fatty acids in animal tissues is not necessarily proportional to their dietary abundance, as it is influenced by their chemical structure and metabolic utilization [36]. This is particularly relevant when evaluating insect-derived ingredients because their lipid composition differs markedly among species. Similarly, cricket meal inclusion had no significant effect on the total cis-monounsaturated fatty acid (MUFA) fraction. This stability is likely attributable to the endogenous regulation of MUFA synthesis via stearoyl-CoA desaturase (SCD), an enzyme that catalyzes the conversion of saturated fatty acids into their corresponding monounsaturated forms to maintain a relatively consistent tissue MUFA composition [60,61]. In contrast, significant differences were observed for odd- and branched-chain fatty acids (OBCFA) and trans-monounsaturated fatty acids (trans-MUFA), although these represented a minor proportion of total lipids. Because odd-chain fatty acids are predominantly of microbial origin, their tissue deposition is often considered an indirect indicator of intestinal microbial activity [62]. Therefore, the reduced OBCFA concentration in ADM rabbits may be associated with the presence of chitin in the cricket meal, which has been reported to influence the intestinal microbial community [63]. However, because gut microbial composition was not evaluated in the present study, this interpretation remains speculative. Conversely, the higher *trans*-MUFA content in ADM rabbits is most likely explained by the presence of trans fatty acid isomers in the cricket meal itself (0.42% of total FAME; Table S1), suggesting a direct dietary contribution to their deposition in muscle.

More pronounced dietary effects were observed within the polyunsaturated fatty acid (PUFA) fraction. Unlike SFA and MUFA, the major PUFA, linoleic acid (18:2 n-6) and α-linolenic acid (18:3 n-3), cannot be synthesized de novo by mammals and therefore depend entirely on dietary supply [64]. Consistent with this, replacing soybean meal with *Acheta domesticus* meal modified the dietary availability of these fatty acids, particularly by reducing the dietary supply of α-linolenic acid (18:3 n-3) and increasing the n-6/n-3 ratio. Although the ADM diet contained a slightly lower proportion of 18:2 n-6, its higher lipid content resulted in a greater overall supply of n-6 fatty acids, contributing to their increased deposition in both muscles. Cricket meal also provided preformed arachidonic acid (20:4 n-6), which may have further contributed to the enrichment of the n-6 fraction. Conversely, the lower dietary supply of α-linolenic acid (18:3 n-3) was reflected in reduced concentrations of total n-3 PUFA in both muscles, including docosapentaenoic acid (DPA; 22:5 n-3) and docosahexaenoic acid (DHA; 22:6 n-3), indicating that even moderate reductions in dietary n-3 availability can influence the deposition of long-chain n-3 PUFA in rabbit meat [60,65]. Furthermore, because the n-6 and n-3 fatty acid families compete for the same elongase and desaturase enzymes [66], the simultaneous increase in n-6 availability and reduction in n-3 substrates likely favored the predominance of the n-6 series. This shift in the balance between n-6 and n-3 PUFA appears to be one of the most relevant biological effects associated with cricket meal inclusion and likely contributed substantially to the separation observed in the PCA. These findings are consistent with the close relationship between dietary lipid composition and the fatty acid profile of rabbit meat, as rabbits incorporate dietary unsaturated fatty acids into tissue lipids with limited biohydrogenation [60]. Importantly, because soybean meal and cricket meal differ in both protein and lipid composition, the independent effects of soybean meal reduction and cricket meal inclusion cannot be completely disentangled. Nevertheless, the present results emphasize that the nutritional evaluation of alternative ingredients should consider not only their protein contribution but also the accompanying changes in dietary lipid composition [61].

The modifications observed in individual fatty acids were clearly reflected in the major fatty acid classes. To date, no studies have evaluated the effects of *Acheta domesticus* meal on the fatty acid composition of rabbit meat. However, studies in other livestock species using insect-derived ingredients support the importance of the dietary lipid source in determining meat fatty acid composition [67]. Diets containing *Hermetia illucens* generally increase SFA deposition and reduce the PUFA fraction [33,56], whereas the inclusion of *Tenebrio molitor* has only minor effects on the major fatty acid classes [31,56]. In contrast, the naturally high α-linolenic acid content of *Bombyx mori* promotes greater n-3 PUFA deposition and a lower n-6/n-3 ratio in rabbit meat [34,57]. Collectively, these studies reinforce the concept that, in rabbits, the fatty acid composition of muscle closely reflects that of the dietary lipid source, with the specific insect species determining the direction and magnitude of the nutritional response.

Among the nutritional lipid indices, dietary treatment significantly affected both the n-6/n-3 and P/S ratios. The higher n-6/n-3 ratio in the ADM group reflected the increase in n-6 PUFA and the concomitant reduction in n-3 PUFA described above. Although a lower n-6/n-3 ratio is generally considered desirable because of the recognized anti-inflammatory and cardioprotective properties of n-3 fatty acids [60], n-6 PUFA also play essential physiological roles by contributing to membrane integrity, growth, immune function, and the synthesis of numerous bioactive lipid mediators [68]. In particular, linoleic acid, which was mainly responsible for the higher n-6 PUFA content observed in ADM-fed rabbits, has been associated with favorable cardiovascular and metabolic outcomes in humans [69]. Conversely, the higher P/S ratio observed in the ADM group indicates a more favorable balance between polyunsaturated and saturated fatty acids. The increase in the P/S ratio resulted primarily from the greater contribution of PUFA rather than from changes in SFA, further supporting the favorable nutritional characteristics of the lipid fraction [60]. Accordingly, neither the atherogenicity index nor the thrombogenicity index was significantly affected by dietary treatment.

Muscle type exerted a major influence on fatty acid composition, affecting most fatty acid classes and nutritional indices. Compared with the LTL, the THM contained higher proportions of SFA and lower proportions of total PUFA and n-3 PUFA despite its greater intramuscular fat content. These differences are consistent with the predominantly oxidative metabolism of the THM, which is more actively involved in locomotion and therefore exhibits greater lipid turnover than the LTL [68,70]. Consequently, the fatty acid profile of the THM likely reflects not only dietary fatty acid supply but also muscle-specific metabolic activity [44,55]. The higher oxidative activity of THM may further contribute to the preferential utilization and turnover of PUFA, particularly long-chain n-3 fatty acids [44,55]. As a consequence, THM exhibited lower percentage of n-3 PUFA and a less favorable n-6/n-3 ratio than LTL. This muscle-dependent metabolism may also explain why dietary effects were generally more pronounced in LTL. Whereas the fatty acid profile of the LTL largely reflects dietary fatty acid supply, the composition of THM appears to be more strongly influenced by local metabolic processes, partially attenuating the differences induced by cricket meal inclusion. These differences were also reflected in the nutritional indices. The higher proportion of saturated fatty acids and lower abundance of n-3 PUFA in THM resulted in higher AI and TI values compared with LTL, although neither index was significantly affected by dietary treatment. Therefore, the observed differences between muscles appear to be primarily associated with their distinct metabolic and physiological roles rather than with diet alone [71,72].

The fatty acid concentrations expressed as mg/100 g of meat largely mirrored the differences in intramuscular fat content between muscles. Accordingly, the THM contained greater amounts of all major fatty acid classes than the LTL, consistent with its higher total lipid content. Therefore, differences in absolute fatty acid concentrations should primarily be interpreted as a consequence of greater fat deposition rather than as evidence of distinct fatty acid metabolism. Nevertheless, when comparing relative proportions (%) with absolute concentrations (mg/100 g), an interesting pattern emerges. Although THM contained larger absolute amounts of all fatty acid classes, the relative distribution of fatty acids differed from that observed in LTL. In particular, the higher fat content of THM was associated with a lower proportional contribution of PUFA, especially n-3 PUFA, and a greater contribution of SFA. This suggests that the additional lipids accumulated in the thigh were mainly deposited as neutral lipids and triglycerides, whereas a relatively greater proportion of long-chain PUFA remained associated with membrane phospholipids, which contribute proportionally more to the leaner LTL muscle [63,73]. Taken together, these results indicate that absolute fatty acid content is largely driven by the amount of intramuscular fat deposited within the muscle, whereas the relative distribution of fatty acid classes reflects the combined effects of lipid deposition and muscle-specific metabolic regulation.

The PCA was performed as an exploratory multivariate approach. Therefore, considering the relatively limited sample size, the multivariate patterns should be interpreted with appropriate caution and regarded as hypothesis-generating rather than confirmatory. Despite these limitations, the PCA highlighted that the response to dietary *Acheta domesticus* meal was mainly associated with modifications in lipid metabolism rather than with changes in productive performance. In particular, variables related to carcass adiposity, together with the most representative fatty acids, contributed to the separation between the dietary treatments. This observation is biologically plausible because, in monogastric species such as rabbits, the fatty acid composition of muscle is strongly influenced by the fatty acid profile of the diet. Moreover, the multivariate findings were consistent with the results of the univariate analyses, providing additional support for the observed treatment-related differences.

## 5. Conclusions

Although the relatively small sample size should be considered, the present study suggests that the partial replacement of soybean meal with *Acheta domesticus* meal could be successfully implemented in diets for growing rabbits without compromising slaughter performance, carcass yield, or the main technological quality traits of meat, supporting the potential of cricket meal as an alternative protein source.

Moreover, no significant sex-related differences were observed in the meat quality traits, carcass characteristics, or fatty acid profiles evaluated in this study. Nevertheless, potential differences between males and females in their response to the greater dietary lipid contribution associated with cricket meal inclusion warrant further investigation, particularly in rabbits slaughtered at later ages, when sex-related differences in lipid deposition may become more pronounced.

The biological response to cricket meal was primarily associated with lipid metabolism rather than carcass performance. Dietary replacement of soybean meal with *Acheta domesticus* modified nutrient partitioning and the fatty acid profile of rabbit meat.

However, the reduction in n-3 PUFA, particularly long-chain n-3 fatty acids, and the consequent increase in the n-6/n-3 ratio indicate a deterioration in the nutritional quality of the lipid fraction, despite the absence of significant changes in other nutritional indices. In addition, the reduction in OBCFA observed in ADM-fed rabbits may be related to the chitin content of the insect meal. Importantly, because cricket meal inclusion coincided with a reduction in soybean meal, the individual contribution of these two dietary changes cannot be fully disentangled within the present experimental design, and their combined effect on lipid deposition and meat fatty acid composition remains to be clarified.

Overall, this study fills an important gap in the current knowledge on the use of *Acheta domesticus* in rabbit nutrition and provides new evidence supporting its potential feed ingredient. Future studies should investigate different inclusion levels and dietary formulations to optimize the nutritional quality of rabbit meat while fully exploiting the potential of cricket meal within sustainable feeding strategies. Although the use of insect-derived processed animal proteins in rabbit diets remains prohibited under the current EU regulatory framework, the present findings contribute to the scientific evidence on their potential use in rabbit nutrition and may support further research aimed at evaluating their safety, nutritional value, and practical applicability in the context of future regulatory developments.

## Figures and Tables

**Table 1:** Analyzed chemical composition (g/100 g diet) and fatty acid profile (% of total FAME) of the experimental diets.

g/100 g of Pellet	CNT	ADM
Dry matter (DM)	93.32	92.51
Ether extract (EE)	2.08	2.64
Crude protein (CP)	17.12	17.46
Crude Fiber (CF)	10.35	10.27
Ash	6.32	6.03
Chitin	-	0.19
N-free extract (NFE) ^1^	57.45	56.11
NDF ^2^	40.31	39.65
ADF ^3^	14.27	14.77
ADL ^4^	2.34	2.47
Digestible Energy (kcal/kg)	2268.68	2270.32
Fatty acids (% of total fatty acid methyl esters)		
16:0	21.11	22.06
18:0	5.83	6.95
18:1	21.25	22.08
18:2 n-6	39.22	37.94
18:3 n-3	7.92	5.27
20:4 n-6	-	0.42
SFA	27.54	29.81
MUFA	23.72	24.78
PUFA n-6	40.82	39.94
PUFA n-3	7.92	5.35
Total PUFA	48.74	45.41
n-6/n-3 ratio	5.15	7.33

CNT: control diet; ADM: diet containing *Acheta domesticus* meal. ^1^ Nitrogen-free extract (NFE) was calculated from DM after subtracting CP, EE, CF, and ash. ^2^ NDF: neutral detergent fiber; ^3^ ADF: acid detergent fiber; ^4^ ADL: acid detergent lignin.

**Table 2:** Ingredient composition of the experimental diets.

Ingredients (%)	CNT	ADM
House cricket (*Acheta domesticus*) meal	-	3.00
Corn grain	20.00	20.00
Barley	18.00	18.00
Soybean meal	8.00	5.00
Wheat bran	15.00	15.00
Alfalfa hay	34.00	34.00
Sunflower oil	1.00	1.00
Cane molasses	2.00	2.00
Calcium carbonate	0.90	0.90
Vitamin–mineral premix ^1^	0.50	0.50
Lysine	0.23	0.23
Methionine + Cysteine	0.15	0.15
L-Threonine	0.07	0.07
Sodium chloride	0.15	0.15

CNT: control diet; ADM: diet containing *Acheta domesticus* meal. ^1^ Vitamin–mineral premix: per kg diet: vitamin A 11,000 IU; vitamin D3 2000 IU; vitamin B1 2.5 mg; vitamin B2 4 mg; vitamin B6 1.25 mg; vitamin B12 0.01 mg; alpha-tocopherol acetate 50 mg; biotin 0.06 mg; vitamin K 2.5 mg; niacin 15 mg; folic acid 0.30 mg; D-pantothenic acid 10 mg; choline 600 mg; Mn 60 mg; Fe 50 mg; Zn 15 mg; I 0.5 mg; Co 0.5 mg.

**Table 3:** Live weight at slaughter, carcass characteristics, and hind leg tissue composition of rabbits fed the experimental diets.

	CNT	ADM	*p* Value
LWS (g)	2325.00 ± 315.13	2486.50 ± 206.84	0.248
CCW (g)	1296.25 ± 168.50	1369.95 ± 127.27	0.079
LvW (g)	83.93 ± 9.26	87.94 ± 14.25	0.158
SFaW (g)	3.15 ± 1.43	6.60 ± 1.54	<0.001
PFaW (g)	8.87 ± 2.44	17.08 ± 3.58	<0.001
FLP (%)	27.78 ± 1.27	27.24 ± 0.55	0.108
TCP (%)	35.74 ± 1.28	36.19 ± 0.72	0.259
HPP (%)	36.48 ± 0.57	36.57 ± 0.59	0.477
HLMeat (%)	75.82 ± 0.72	74.97 ± 0.59	0.047
HLBone (%)	21.81 ± 0.67	22.00 ± 0.96	0.705
HLFat (%)	2.37 ± 0.55	3.04 ± 0.84	0.011

CNT: control diet; ADM: diet containing *Acheta domesticus* meal. LWS: Live Weight at slaughter; CCW: chilled carcass weight; LvW: liver weight; SFaW: scapular fat weight; PFaW: dissectible perirenal and pelvic fat weight; FLP: foreleg part; TCP: thoracic cage part; HPP: hind part; HLMeat: hind leg dissectible meat; HLBone: hind leg dissectible bone; HLFat: hind leg dissectible fat and connective tissue proportion. Values are expressed as mean ± standard deviation.

**Table 4:** Physical characteristics of the *Longissimus thoracis et lumborum* muscle of rabbits fed the experimental diets.

Parameter	CNT	ADM	*p* Value
pH	5.89 ± 0.17	5.81 ± 0.11	0.152
WHC %	8.01 ± 1.90	7.78 ± 1.47	0.980
Cooking loss %	26.62 ± 2.04	28.65 ± 1.77	0.039
L*	50.06 ± 1.73	49.81 ± 3.40	0.922
a*	−3.32 ± 0.51	−2.99 ± 0.68	0.371
b*	3.65 ± 1.12	3.53 ± 1.79	0.896

CNT: control diet; ADM: diet containing *Acheta domesticus* meal. WHC: water-holding capacity; L*: lightness; a*: a index, redness; b*: b index, yellowness. Values are expressed as mean ± standard deviation.

**Table 5:** Major fatty acid classes (% of total FAME) in the *Longissimus thoracis et lumborum* (LTL) and thigh muscles (THM) of rabbits fed the experimental diets.

	LTL			THM				
	CNT	ADM	Sign	CNT	ADM	Sign	*p* Value Diets	*p* Value Muscles
SFA	38.14 ± 1.22	37.53 ± 0.74	ns	40.39 ± 1.22	39.39 ± 0.92	ns	0.068	<0.001
OBCFA	1.76 ± 0.11	1.21 ± 0.14	***	1.90 ± 0.22	1.79 ± 0.11	**	<0.001	<0.001
MUFAc	31.09 ± 1.41	30.90 ± 1.89	ns	29.74 ± 1.07	29.71 ± 2.28	ns	0.458	0.223
MUFAt	0.15 ± 0.02	0.23 ± 0.03	***	0.18 ± 0.06	0.21 ± 0.04	**	<0.001	<0.001
PUFA n-6	24.55 ± 2.26	27.05 ± 1.57	***	24.08 ± 1.47	25.75 ± 1.71	*	0.013	0.005
PUFA n-3	3.58 ± 0.22	2.40 ± 0.11	***	2.80 ± 0.42	2.25 ± 0.30	**	<0.001	0.002
Total PUFA	28.35 ± 2.35	30.11 ± 1.58	*	27.24 ± 1.16	28.40 ± 1.75	*	0.047	<0.001
n-6/n-3 ratio	6.85 ± 0.59	11.27 ± 0.69	***	8.60 ± 1.65	11.44 ± 1.01	***	<0.001	<0.001
P/S ratio	0.54 ± 0.06	0.61 ± 0.03	*	0.58 ± 0.05	0.63 ± 0.04	*	0.026	0.087
AI	0.58 ± 0.06	0.56 ± 0.02	ns	0.66 ± 0.03	0.65 ± 0.02	ns	0.194	0.014
TI	0.93 ± 0.05	0.97 ± 0.08	ns	1.12 ± 0.05	1.10 ± 0.03	ns	0.056	0.043

CNT: control diet; ADM: diet containing *Acheta domesticus* meal. SFA: saturated fatty acids; OBCFA: odd- and branched-chain fatty acids; MUFAt: *trans*-monounsaturated fatty acids; MUFAc: *cis*-monounsaturated fatty acids; PUFA n-6: omega-6 polyunsaturated fatty acids; PUFA n-3: omega-3 polyunsaturated fatty acids; PUFA: total polyunsaturated fatty acids; AI: atherogenicity index = (12:0 + 4 × 14:0 + 16:0)/(MUFA + PUFA); TI: thrombogenicity index = (14:0 + 16:0 + 18:0)/(0.5 × MUFA + 0.5 × PUFA n-6 + 3 × PUFA n-3 + n-3/n-6); P/S = (18:2 n-6 + 18:3 n-3)/(12:0 + 14:0 + 16:0 + 18:0). Values are expressed as mean ± standard deviation. Significance of dietary treatment within each muscle type is reported in the “Sign” column (ns = not significant; * *p* < 0.05; ** *p* < 0.01; *** *p* < 0.001). The last two columns indicate the significance of the main effects of diet and muscle.

**Table 6:** Major fatty acid classes (mg/100 g of meat) in the *Longissimus thoracis et lumborum* (LTL) and thigh muscles (THM) of rabbits fed the experimental diets.

	LTL			THM				
	CNT	ADM	Sign	CNT	ADM	Sign	*p* Value Diets	*p* Value Muscles
SFA	296.65 ± 38.46	317.56 ± 42.66	ns	716.52 ± 73.58	734.65 ± 57.90	ns	0.055	<0.001
OBCFA	12.65 ± 1.43	11.45 ± 2.65	ns	33.02 ± 4.89	32.83 ± 2.04	ns	0.820	<0.001
MUFAc	245.60 ± 18.25	279.92 ± 26.63	**	531.66 ± 63.03	594.56 ± 67.92	**	0.012	<0.001
MUFAt	3.53 ± 0.26	4.68 ± 0.61	**	10.15 ± 1.40	11.42 ± 1.13	*	0.011	<0.001
PUFA-n-6	194.43 ± 22.75	242.96 ± 32.57	***	431.89 ± 40.38	467.82 ± 42.55	*	0.009	<0.001
PUFA n-3	29.64 ± 2.58	21.64 ± 2.49	***	57.16 ± 6.23	52.29 ± 4.75	*	0.006	<0.001
Total PUFA	224.07 ± 24.49	264.60 ± 34.85	**	489.05 ± 42.83	520.11 ± 44.41	*	0.028	<0.001

CNT: control diet; ADM: diet containing *Acheta domesticus* meal. SFA: saturated fatty acids; OBCFA: odd chain and branched fatty acids; MUFAt: trans monounsaturated fatty acids; MUFAc: cis monounsaturated fatty acids; PUFA n-6: omega-6 polyunsaturated fatty acids; PUFA n-3: omega-3 polyunsaturated fatty acids; PUFA: total polyunsaturated fatty acids. Values are expressed as mean ± standard deviation. Significance of dietary treatment within each muscle type is reported in the “Sign” column (ns = not significant; * *p* < 0.05; ** *p* < 0.01; *** *p* < 0.001). The last two columns indicate the significance of the main effects of diet and muscle.

## Data Availability

The original contributions presented in the study are included in the article and Supplementary Materials.

## Supplementary Materials

The following supporting information can be downloaded at https://www.mdpi.com/article/10.3390/ani16162596/s1: Table S1: Chemical composition, amino acid profile, and fatty acid profile of soybean meal and *Acheta domesticus* meal used in the experimental diets [61,74]; Table S2: Live weight at slaughter, carcass characteristics, and hind leg tissue composition of rabbits (males and females) fed the experimental diets; Table S3: Proximate composition of the *Longissimus thoracis et lumborum* (LTL) and thigh muscles (THM) of rabbit (males and females) fed the experimental diets; Table S4: Individual fatty acids expressed as percentage of total fatty acid methyl esters (FAME) in the *Longissimus thoracis et lumborum* (LTL) and thigh muscles (THM) of rabbits (males and females fed the experimental diets; Table S5: Loadings of the variables included in the principal component analysis (PCA).

## Abbreviations

The following abbreviations are used in this manuscript:

AA	Arachidonic Acid
ADF	Acid Detergent Fiber
ADL	Acid Detergent Lignin
ADM	Diet containing *Acheta domesticus* meal
AI	Atherogenicity index
ALA	α-linolenic acid
CCW	Chilled carcass weight
CF	Crude Fiber
CNT	Control diet
CP	Crude Protein
DE	Digestible Energy
DM	Dry Matter
DPA	Docosapentaenoic acid
DHA	Docosahexaenoic acid
EE	Ether Extract
EPA	Eicosapentaenoic acid
FAME	Fatty acid methyl esters
FLP	Foreleg part
GC-FID	Gas Chromatography–Flame Ionization Detection
HL	Hind leg
HPP	Hind Part
IUPAC	International Union of Pure and Applied Chemistry
LA	Linoleic acid
LTL	Longissimus thoracis et lumborum muscle
LWS	Live Weight at Slaughter
LvW	Liver Weight
MUFA	Monounsaturated fatty acids
MUFAc	Cis-Monounsaturated Fatty Acids
MUFAt	Trans-Monounsaturated Fatty Acids
NDF	Neutral Detergent Fiber
NFE	Nitrogen-Free Extract
OBCFA	Odd- and Branched-Chain Fatty Acids
P/S	Polyunsaturated-to-Saturated Fatty Acid Ratio
PAPs	Processed Animal Proteins
PCA	Principal Component Analysis
PFaW	Perirenal-Pelvic Fat Weight
PUFA	Polyunsaturated fatty acids
SCD	Stearoyl-CoA Desaturase
SFA	Saturated fatty acids
SFaW	Scapular Fat Weight
TCP	Thoracic Cage Part
THM	Thigh muscle
TI	Thrombogenicity index
WHC	Water-Holding Capacity
