# Supplementary material for: Effect of Dietary Inclusion of House Cricket (Acheta domesticus) Meal on Carcass Characteristics and Meat Quality in Rabbits
Source: Animals (Basel). 2026 Aug 19;16(16):2596. doi: 10.3390/ani16162596 (PMC13509785; doi:10.3390/ani16162596)
Supplement: Supplementary file 1 [file animals-16-02596-s001.zip › animals-4447559-supplementary.pdf]

Table S1. Chemical composition, amino acid profile, and fatty acid profile of soybean meal and *Acheta domesticus* meal used in the experimental diets.

Parameter (g/100 g)	Soybean meal	*Acheta domesticus* meal
**Chemical composition**		
Dry matter	88.51	89.19
Ash	6.42	6.36
Crude fat	2.17	18.54
Crude protein	49.23	59.72
Crude fiber	6.48	-
Chitin	-	4.46
**Amino acid composition**[¥]		
Lys+ Cys	3.31	3.57
Met	0.65	1.33
Thr	1.97	3.06
Try	0.68	1.91
Val	2.33	4.69
Leu	3.64	4.86
Ile	2.17	2.58
His	1.34	2.18
Phe	2.58	4.03
Arg	3.62	4.19
Ala+ Asp	5.52	3.68
Glu	8.41	8.04
Pro	2.76	3.57
Serine	2.45	3.68
**Fatty acid profile (% of total FAME)**		
14:0	0.21	0.43
16:0	14.38	16.12
16:1 cis-9	0.15	0.48
18:0	5.92	9.63
18:1 cis-9	20.73	22.31
18:1 cis-11	0.45	1.23
18:2 n-6 (LA)	50.71	43.86
20:0	0.23	0.27
20:1	0.21	0.30
18:3 n-3 (ALA)	7.01	3.72
20:2 n-6	-	0.54
20:4 n-6 (AA)	-	0.84
20:5 n-3 (EPA)	-	0.27
∑SFA	20.74	26.45
∑MUFA cis	21.54	24.32
∑PUFA n-6	50.71	45.24
∑PUFA n-3	7.01	3.99
∑PUFA	57.72	49.23
∑TRANS isomers	-	0.42
n-6/n-3 ratio	7.23	12.96

DM = dry matter; Lys = lysine; Cys = cysteine; Met = methionine; Thr = threonine; Try = tryptophan; Val = valine; Leu = leucine; Ile = isoleucine; His = histidine; Phe = phenylalanine; Arg = arginine; Ala = alanine; Asp = aspartic acid; Glu = glutamic acid; Pro = proline. FAME = Fatty acid methyl esters; LA = linoleic acid (18:2n-6); ALA = α-linolenic acid (18:3n-3); AA = arachidonic acid (20:4n-6); EPA = eicosapentaenoic acid (20:5n-3). SFA = saturated fatty acids; MUFA = monounsaturated fatty acids; PUFA = polyunsaturated fatty acids. The n-6/n-3 ratio was calculated as the ratio between total n-6 and total n-3 polyunsaturated fatty acids.
[¥] The amino acid composition was determined by HPLC following the extraction procedure described by Fehri et al. (2025) [1], whereas amino acid derivatization with dansyl chloride and the subsequent HPLC analysis were performed according to the method of Navarro-Abril et al. (2022) [2].

Table S2. Live weight at slaughter, carcass characteristics, and hind leg tissue composition of rabbits (males and females) fed the experimental diets.

	CNT			ADM				
	Males	Females	*Sig.*	Males	Females	*Sign.*	***p* value diets**	***p* value sexes**
LWS (g)	2329.6±318.3	2311.4±284.2	ns	2492.0±301.2	2470.2±227.4	ns	0.342	0.882
CCW (g)	1296.9±193.6	1294.6±103.8	ns	1379.7±145.6	1340.7±80.4	ns	0.193	0.780
LvW (g)	84.34±10.41	82.83±6.85	ns	88.83±15.59	85.27±11.41	ns	0.572	0.679
SFaW (g)	3.24±1.64	2.9±0.87	ns	6.67±1.77	6.4±0.86	ns	<0.001	0.688
PFaW (g)	8.98±2.85	8.60±1.05	ns	16.93±3.94	17.50±2.82	ns	<0.001	0.851
FLP (%)	27.91±1.48	27.41±0.23	ns	27.25±0.52	27.20±0.75	ns	0.372	0.569
TCP (%)	35.64±1.50	36.05±0.34	ns	36.07±0.73	36.56±0.68	ns	0.354	0.377
HPP (%)	36.46±0.67	36.54±0.23	ns	36.68±0.63	36.24±0.36	ns	0.780	0.532
HLMeat (%)	75.84±0.85	75.78±0.30	ns	75.03±0.69	74.76±0.47	ns	0.031	0.616
HLBone (%)	21.72±0.74	22.03±0.52	ns	21.87±0.97	22.38±0.92	ns	0.546	0.326
HLFat (%)	2.44±0.63	2.19±0.23	ns	3.10±0.91	2.86±0.73	ns	0.077	0.502

CNT: control diet; ADM: diet containing *Acheta domesticus* meal. LWS: Live Weight at slaughter; CCW: chilled carcass weight; LvW: liver weight; SFaW: scapular fat weight; PFaW: dissectible perirenal and pelvic fat weight; FLP: foreleg part; TCP: thoracic cage part; HPP: hind part; HLMeat: hind leg dissectible meat; HLBone: hind leg dissectible bone; HLFat: hind leg dissectible fat and connective tissue proportion. Values are expressed as mean ± standard deviation.

Table S3. Proximate composition of the *Longissimus thoracis et lumborum* (LTL) and thigh muscles (THM) of rabbit (males and females) fed the experimental diets.

	**LTL**			**THM**					
	**CNT**	**ADM**	**Sign**	**CNT**	**ADM**	**Sign**	***p* value diets**	***p* value muscles**	***p* value sexes**
DM	23.47±0.61	23.85±0.56	ns	23.87±0.87	24.16±0.59	ns	0.187	0.265	0.201
Ash	1.30±0.02	1.31±0.06	ns	1.30±0.03	1.29±0.04	ns	0.161	0.138	0.432
Protein	21.22±0.63	21.47±0.51	ns	20.40±0.66	20.48±0.31	ns	0.104	0.092	0.672
Fat	0.95±0.06	1.07±0.11	*	2.17±0.24	2.39±0.20	*	0.017	0.006	0.139

CNT: control diet; ADM: diet containing *Acheta domestics* meal. DM: dry matter. Data are presented as mean ± standard deviation. Significant differences between diets and

between muscles are reported as *p<0.05; **p<0.01. The last three columns indicate the significance of the main effects of diet, muscle and sex.

Table S4. Individual fatty acids expressed as percentage of total fatty acid methyl esters (FAME) in the *Longissimus thoracis et lumborum* (LTL) and thigh muscles (THM) of rabbits (males and females) fed the experimental diets.

	LTL			THM					
	CNT	ADM	Sign	CNT	ADM	Sign	*p* value diets	*p* value muscles	*p* value sexes
12:0	0.10±0.02	0.08±0.02	ns	0.10±0.03	0.09±0.03	ns	0.120	0.387	0.454
14:0	1.84±0.45	1.74±0.23	ns	2.17±0.14	2.02±0.15	ns	0.616	<0.001	0.058
14:1	0.15±0.04	0.15±0.05	ns	0.16±0.03	0.17±0.02	ns	0.428	0.459	0.283
15:0 anteiso	0.10±0.02	0.08±0.01	**	0.12±0.02	0.10±0.01	*	0.006	<0.001	0.076
15:0	0.51±0.04	0.43±0.06	**	0.56±0.06	0.52±0.03	*	0.002	<0.001	0.361
15:1	0.14±0.04	0.13±0.03	ns	0.15±0.04	0.17±0.04	ns	0.647	0.004	0.122
16:0	26.89±1.05	26.76±0.61	ns	28.08±0.79	27.83±0.58	ns	0.741	<0.001	0.855
16:1 n-7	0.32±0.09	0.33±0.03	ns	0.32±0.02	0.30±0.04	ns	0.579	0.486	0.435
16:1 n-9	3.30±0.71	3.24±1.08	ns	3.26±0.45	3.15±0.74	ns	0.147	0.363	0.169
17:0	0.67±0.03	0.57±0.06	***	0.74±0.11	0.68±0.07	**	<0.001	<0.001	0.890
17:1	0.34±0.06	0. 30±0.07	ns	0.33±0.06	0.32±0.04	ns	0.074	0.121	0.310
18:0	7.81±0.52	7.67±0.34	ns	8.80±0.55	8.34±0.50	ns	0.081	<0.001	0.779
18:1trans	0.10±0.02	0.14±0.03	ns	0.18±0.03	0.19±0.03	ns	0.084	<0.001	0.523
18:1 n-9	22.180.85±	22.24±0.81	ns	21.82±0.64	22.03±1.73	ns	0.332	0.502	0.493
18:1 n-7	2.72±0.26	2.50±0.22	*	2.55±0.24	2.53±0.19	ns	0.043	0.087	0.419
18:2 trans	0.12±0.03	0.15±0.04	*	0.31±0.05	0.34±0.08	*	0.041	<0.001	0.181
18:2 n-6	17.92±1.55	20.72±1.24	***	20.49±1.48	22.53±1.29	**	0.007	0.002	0.150
20:0	0.17±0.04	0.15±0.03	*	0.11±0.02	0.11±0.02	ns	0.042	0.038	0.282
20:1	0.20±0.02	0.21±0.03	ns	0.21±0.03	0.21±0.02	ns	0.876	0.355	0.699
18:3 n-3	1.76±0.17	1.27±0.09	***	1.98±0.30	1.68±0.23	**	0.006	<0.001	0.713
CLA	0.10±0.02	0.11±0.02	ns	0.05±0.02	0.06±0.03	ns	0.105	0.007	0.599
20:2 n-6	0.13±0.02	0.14±0.02	ns	0.20±0.02	0.22±0.02	*	0.027	<0.001	0.325
22:0	0.66±0.10	0.56±0.07	***	0.39±0.05	0.32±0.09	**	0.002	<0.001	0.416
20:4 n-6	5.78±0.77	5.84±0.46	ns	3.00±0.23	2.61±0.97	ns	0.356	<0.001	0.325
22:2 n-6	0.17±0.04	0.17±0.02	ns	0.09±0.03	0.08±0.03	ns	0.542	<0.001	0.361
20:5 n-3	0.17±0.05	0.15±0.02	ns	0.08±0.02	0.07±0.02	ns	0.064	<0.001	0.712
24:0	1.37±0.25	1.42±0.17	ns	0.75±0.05	0.67±0.22	ns	0.084	<0.001	0.149
22:4 n-6	0.55±0.10	0.58±0.06	ns	0.30±0.03	0.31±0.09	ns	0.072	<0.001	0.541
22:5 n-3	1.36±0.15	0.81±0.07	***	0.63±0.15	0.42±0.18	**	<0.001	<0.001	0.416
22:6 n-3	0.29±0.07	0.17±0.03	***	0.11±0.03	0.08±0.03	**	<0.001	<0.001	0.537
SFA	38.14±1.22	37.53±0.74	ns	40.39±1.22	39.39±0.92	ns	0.102	<0.001	0.506
MUFA	31.09±1.41	30.90±1.89	ns	29.74±1.07	29.71±2.28	ns	0.279	0.103	0.261
PUFAn-6	24.55±2.26	27.05±1.57	**	24.08±1.47	25.75±1.71	*	0.021	0.005	0.112
PUFAn-3	3.58±0.22	2.40±0.11	***	2.80±0.42	2.25±0.30	**	<0.001	0.012	0.588
n-6/n-3	6.85±0.59	11.27±0.69	***	8.60±1.65	11.44±1.01	**	<0.001	0.002	0.379

CNT: control diet; ADM: diet containing *Acheta domestics* meal. Data are presented as mean ±SD standard deviation. Significance of dietary treatment within each muscle type

is reported in the "Sign" column (ns = not significant; *p<0.05; **p<0.01; ***p<0.001). The last three columns indicate the significance of the main effects of diet, muscle and sex.

Table S5. Loadings of the variables included in the principal component analysis (PCA)

	PC1	PC2
CCW	0.0691	-0.0442
PFaW	0.5378	-0.1526
SFaW	0.6055	-0.4956
L*	0.0017	0.0248
a*	-0.0639	0.0651
b*	-0.0897	0.2084
Cooking loss%	0.0670	-0.0343
Fat %	0.0685	0.1011
Protein %	-0.0849	-0.0302
16:0 %	0.0072	0.0545
18:0 %	0.0094	-0.0964
17:0 %	-0.0959	-0.0606
16:1 cis-9 %	-0.0243	0.3304
18:1cis-9 %	0.0043	0.0585
18:2 n-6 %	0.0932	-0.0720
18:3 n-3 %	-0.1724	-0.0269
20:4 n-6 %	0.0192	-0.1483
20:5 n-3 %	-0.3489	-0.4571
22:5 n-3 %	-0.2755	-0.3505
22:6 n-3 %	-0.2800	-0.4730

Performance data: CCW: chilled carcass weight; SFaW: scapular fat weight; PFaW: dissectible perirenal and pelvic fat weight; phisical quality on *Longissimus thoracis et lumborum:* L*, a*, b*, cooking loss%; Proximate composition: fat%, protein %, principal fatty acids considering saturated fatty acids, monounsaturated fatty acids, polyunsaturate fatty acids and long chain fatty acids.

1. Fehri, N.E.; Contò, M.; Castrica, M.; Quattrone, A.; Renzi, G.; Di Giovanni, S.; Agradi, S.; Vigo, D.; Brecchia, G.; Menchetti, L.; et al. Effects of Diets Containing Extruded Linseed and Padina Pavonica Algae on Meat Rabbit: Carcass Performance and Meat Quality. *Foods* **2025**, *14*, 274, doi:10.3390/foods14020274.
2. Navarro-Abril, A.; Saurina, J.; Sentellas, S. Simultaneous Determination of Amino Acids and Biogenic Amines by Liquid Chromatography Coupled to Mass Spectrometry for Assessing Wine Quality. *Beverages* **2022**, *8*, 69, doi:10.3390/beverages8040069.
